# A Self‐Assemble Supramolecular Film with Humidity Visualization Enabled by Clusteroluminescence

**DOI:** 10.1002/advs.202304946

**Published:** 2023-11-09

**Authors:** Xiang Chen, Chenxi Hu, Yang Wang, Ting Li, Jie Jiang, Jing Huang, Shibo Wang, Weifu Dong, Jinliang Qiao

**Affiliations:** ^1^ The Key Laboratory of Synthetic and Biological Colloids Ministry of Education School of Chemical and Material Engineering Jiangnan University 1800 Lihu Road Wuxi 214122 China; ^2^ SINOPEC Beijing Research Institute of Chemical Industry Beijing 100013 China

**Keywords:** clusteroluminescence, dynamic bond, self‐assemble, stimuli‐responsive materials

## Abstract

Clusteroluminescence (CL) has recently gained significant attention due to its unique through‐space interactions associated with a high dependence on the aggregation of subgroups. These distinct features could easily transform the stimuli into visual fluorescence and monitor the fluctuation of the environment but have not received sufficient attention before. In this work, supramolecular films are designed based on the neutralization reaction of anhydride groups and the self‐assembly of dynamic covalent disulfide bonds in NaOH aqueous solution. The self‐assembly of hydrophilic carboxylate chromophores and hydrophobic disulfide‐containing five‐membered rings could be observed by the variation of the aggregation state of carboxylate in CL. Furthermore, the dynamic cross‐linking films obtained with water‐sensitive carboxylate chromophores could alter the aggregation distance stimulated by surrounding water vapor, causing the emission wavelength to change from 534 to 508 nm by varying the relative humidity. This work not only provides an approach to monitor the self‐assembly of clusteroluminogens but also offers new strategies for designing stimuli‐responsive materials that utilize the intrinsic features of CL.

## Graphical abstract

The dependence of clusteroluminescence (CL) toward the aggregation of subgroups allows the visualization of stimuli via fluorescence, and the responsive CL is realized by the stimuli of aggregation during the self‐assembly and the stimuli of vapor under different relative humidities through emission intensity and emission wavelength, respectively.

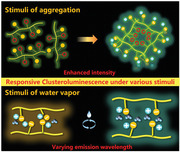

## 1 Introduction

Organic fluorescent materials have been widely used in our daily life,^[^ ^1^ ^]^ but traditional fluorescent materials with large π‐conjugated structures are susceptible to aggregation‐caused quenching. New organic fluorescent with special aggregation‐induced emission properties have been discovered by scientists.^[^ ^2^ ^]^ However, the complex synthesis of large π‐conjugated structure and underlying biotoxicity of polycyclic aromatic structure still hinder the practical application.^[^ ^3^ ^]^

Recently, a series of fluorescent materials without traditional large π‐conjugated structures have emerged, such as copolymers of maleic anhydride, phenolic resins, silicone surfactant, protein, starch, and polyester possessing ether, carbonyl, cyano, hydroxyl, thioether, and amide subgroups.^[^ ^4^ ^]^ They are able to emit fluorescence in the clustering state of electron‐rich subgroups, known as clusteroluminescence (CL), despite the absence of large‐conjugated structures. The through‐space interaction (TSI) mechanism, which requires the aggregation of subgroups, was proposed to explain CL.^[^ ^5^ ^]^ Therefore, the precise design of subgroups that are sensitive to environmental stimuli could change the aggregate states upon stimuli and provide a direct visualization way to convert stimuli into optical CL.^[^ ^6^ ^]^ Since there is no traditional large π‐conjugated structure, clusteroluminogens (CLgens) always feature simple synthesis, low biotoxicity, and species diversity, which could generate a huge breakthrough in flexible and wearable materials. However, the applications of the intrinsic properties of CL are rarely reported.^[^ ^7^ ^]^

Poly(maleic anhydride‐*alt*‐vinyl acetate) (PMV) is a well‐known CLgens with inexpensive monomers and a simple synthetic route. By controlling the concentration of NaOH in aqueous solution, the emission wavelength of PMV can be easily tuned.^[^ ^8^ ^]^ However, as the source of the CL, the water sensitivity of the carboxylate group is overlooked. Since TSI is closely related to the aggregation distance of subgroups, the water‐sensitive carboxylate group could be perfectly served at a humidity response medium. Upon the stimuli of the surrounding water vapor, the aggregation distance of the carboxylate group would induce the variation of TSI, leading to the response in emission wavelength.

Lipoic acid (LA) is a naturally occurring molecule, whose dynamic disulfide‐containing five‐membered ring could introduce cross‐linking structure in flexible and wearable materials.^[^ ^9^ ^]^ Interestingly, LA also exhibits distinct evaporation‐induced interfacial self‐assembly in NaOH with subsequently dynamic covalent ring‐opening‐polymerization (ROP).^[^ ^10^ ^]^ It remains unclear whether and how the dynamic disulfide‐containing five‐membered ring can self‐assemble in supramolecules, which could provide a new conceptual strategy for designing recyclable polymeric materials.

Herein, we provide a visual sensing approach based on the changing aggregation states of subgroups. As detailed below, we designed an ethylenediamine‐modified LA that could easily react with the anhydride group in PMV. By dissolving PMV‐LA in NaOH aqueous, we were able to implement both the introduction of the carboxylate group and the self‐assembly of the hydrophilic carboxylate group and hydrophobic disulfide‐containing five‐membered ring. This allowed us to monitor the self‐assembly process by tracking the variation of aggregation of the carboxylate group. Furthermore, the intermolecular dynamic covalent exchange of gathering disulfide bonds realizes the ROP, resulting in relatively stable supramolecular films with self‐healing and reprocessing properties. The CL of dual emission region films could be easily tuned by adjusting the grafting ratio and NaOH amounts. Meanwhile, the water‐sensitive carboxylate group could change its aggregation distance stimulated by surrounding water vapor. As a result, the supramolecular films exhibited a change in emission wavelength from 534 to 508 nm by varying the relative humidity (RH) from 10% to 90%, showing a good linear relationship. We foresee that the synergistic strategy of dynamic covalent and noncovalent ionic bonds offers a new perspective for developing humidity sensors based on the distinct features of TSI.

## 2 Results and Discussion

**Figure 1:**
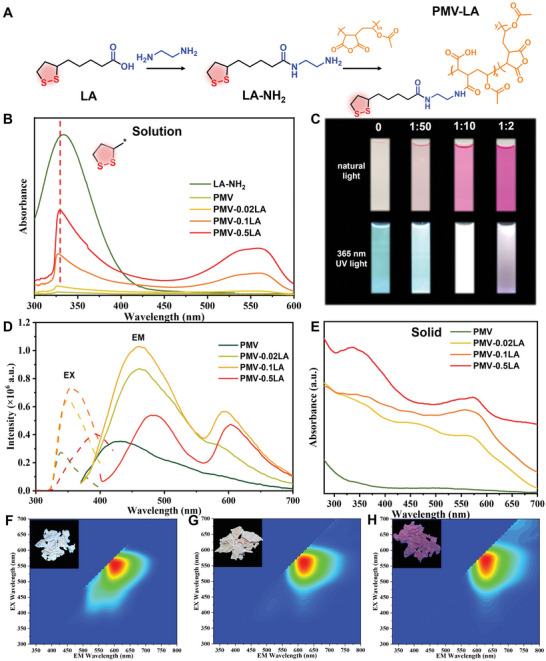
A) Conceptual illustration of the synthesis of LA‐NH_2_ and PMV‐LA. B) UV–vis absorption spectra of LA‐NH_2_, PMV, PMV‐0.02LA, PMV‐0.1LA, and PMV‐0.5LA solutions in acetone (5 mg mL^−1^). C) Photographs of PMV, PMV‐0.02LA, PMV‐0.1LA, and PMV‐0.5LA solutions under natural and UV light. D) Photoluminescence spectra of PMV, PMV‐0.02LA, PMV‐0.1LA, and PMV‐0.5LA solutions in acetone (5 mg mL^−1^). E) The UV–vis diffuse reflection spectra of PMV, PMV‐0.02LA, PMV‐0.1LA and PMV‐0.5LA solids. Normalized 3D photoluminescence spectra of F) PMV‐0.02LA, G) PMV‐0.1LA, and H) PMV‐0.5LA solids. (Insets: Photographs of PMV‐0.02LA, PMV‐0.1LA, and PMV‐0.5LA solids under UV light).

As shown in **Figure** **1**A, LA‐NH_2_ was synthesized by the amide reaction of LA and ethylenediamine.^[^ ^11^ ^]^ Fourier transform infrared (FTIR) and ^1^H NMR were utilized to confirm the successful synthesis of LA‐NH_2_. The disappearance of the peak at 1685 cm^−1^ in LA and the new absorption peak at 1641 cm^−1^ and 1538 cm^−1^ in LA‐NH_2_ verified the changes from ─COOH to ─CONH─ (Figure S1, Supporting Information). The ^1^H NMR of LA‐NH_2_ in Figure S2, Supporting Information supported this as well. Next, the single primary amine in LA‐NH_2_ easily reacted with the anhydride group in PMV synthesized by self‐stabilized precipitation polymerization, which was also confirmed by FTIR and ^1^H NMR. After ring‐opening, the stretching vibrations of C═O in anhydride groups at around 1779 cm^−1^ weakened, while the newly formed ─COOH and ─CONH─ at 1706, 1657, and 1540 cm^−1^ became stronger with increased addition of LA‐NH_2_ (Figure S3, Supporting Information). Besides, the H (a) atom in the disulfide‐containing five‐membered ring could also be observed in PMV‐LA (Figure S4, Supporting Information), and the ratio of the integral area of H (a) to H (4) was similar to the adding amount. The obtained polymer was named PMV‐xLA, with x representing the ratio of PMV repeating unit to LA‐NH_2_. The UV–vis spectrum of their solution also showed an enhanced absorbance peak at 330 nm, which reflects the disulfide‐containing five‐membered ring (Figure 1B).

Interestingly, the color of PMV‐LA solutions and solids changed significantly from PMV to PMV‐0.5LA, which motivated us to study their optical properties (Figure S5, Supporting Information and Figure 1C). The UV–vis spectrum of PMV‐LA showed a new absorption peak at 550 nm, and the photoluminescence spectra exhibited an additional peak at around 600 nm, which became more obvious with increased LA‐NH_2_ addition (Figure 1B,D). These changes in absorption and emission spectra were consistent with the observations under natural light and UV light. Similarly, the UV–vis diffuse reflection spectra of PMV‐LA solids displayed a new absorption peak at 570 nm (Figure 1E). The 3D emission spectra (Figure 1F–H) and photoluminescent spectra (Figure S6, Supporting Information) of PMV‐LA solids showed red‐shifted emission, with the maximum emission wavelength changing from 610 to 630 nm (Table S1, Supporting Information). The average lifetimes of PMV and PMV‐LA solids at 610 nm also varied (Figure S7, Supporting Information). These observations suggest the formation of new chromophores in PMV‐LA, which may be attributed to the introduction of electron‐rich atoms in the anhydride group, facilitating TSI of subgroups.^[^ ^12^ ^]^ Considering that most CLgens emit in the blue region, this amide reaction could serve as a new approach to realize the red emission of maleic anhydride copolymers.

**Figure 2:**
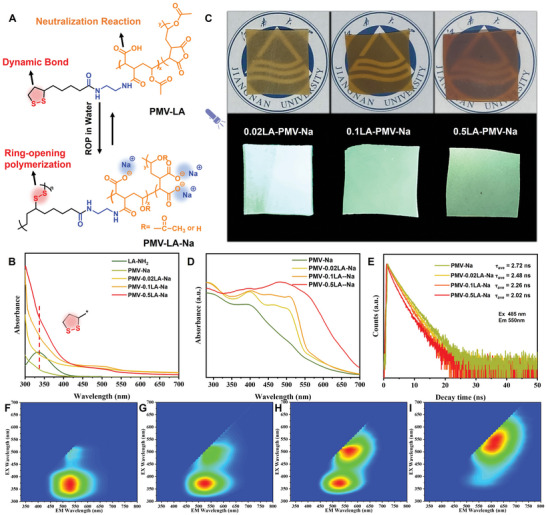
A) Conceptual illustration of the synthesis of PMV‐LA‐Na. B) UV–vis absorption spectra of LA‐NH_2_, PMV‐Na, PMV‐0.02LA‐Na, PMV‐0.1LA‐Na, and PMV‐0.5LA‐Na precursor solutions. C) Photographs of PMV‐0.02LA‐Na, PMV‐0.1LA‐Na, and PMV‐0.5LA‐Na films under natural and UV light. D) The UV–vis diffuse reflection spectra of PMV‐Na, PMV‐0.02LA‐Na, PMV‐0.1LA‐Na and PMV‐0.5LA‐Na films. E) Decay times spectra of PMV‐Na, PMV‐0.02LA‐Na, PMV‐0.1LA‐Na, and PMV‐0.5LA‐Na films excited at 485 nm. Normalized 3D photoluminescence spectra of F) PMV‐Na, G) PMV‐0.02LA‐Na, H) PMV‐0.1LA‐Na, and I) PMV‐0.5LA‐Na films.

As previously reported, the evaporation‐induced interfacial supramolecular self‐assembly could realize the ROP of LA in NaOH aqueous solution.^[^ ^10^, ^13^ ^]^ This is due to the assembly of amphiphilic small molecules during the evaporation of water, leading to a structurally ordered layered network. This process is similar to the preparation of nacre‐like composites with typical brick‐and‐mortar architecture.^[^ ^14^ ^]^ The equilibrium states of the dynamic self‐assembly process strongly depend on concentration, which can organize the building blocks and induce the subsequent polymerization. Meanwhile, PMV also showed intriguing CL after the ring‐opening of the anhydride group in NaOH aqueous solution. It remains unclear whether the evaporation process would induce the ROP of a side‐chain disulfide‐containing five‐membered ring in supramolecules, which could attach intrinsic dynamicity to CL polymers and broaden their applications (**Figure** **2**A).

The NaOH aqueous solutions of PMV‐LA also showed an increased grafting ratio of LA‐NH_2_, with enhanced absorbance at around 330 nm (Figure 2B). After evaporation at room temperature, PMV‐LA‐Na films with green CL under UV light and a cross‐linking structure that only swelled in water were obtained (Figure 2C and Figure S8, Supporting Information). Then, the thermal properties of PMV‐LA‐Na film were investigated (Figure S9, Supporting Information). The glass transition temperature (*T*_g_) of PMV‐LA‐Na initially increased and then decreased with the addition of LA‐NH_2_. The higher *T*_g_ of PMV‐0.1LA‐Na was attributed to the cross‐linking structure, while the decreased *T*_g_ of PMV‐0.02LA‐Na and PMV‐0.5LA‐Na was due to the introduction of flexible side chains, as evidenced by the lower *T*_g_ of pLA‐NH_2_ (pLA‐NH_2_ was prepared by the evaporation of solvents of LA‐NH_2_, which will be discussed later). Moreover, the optical properties of PMV‐LA‐Na also changed with the increased addition of LA‐NH_2_. The 3D emission spectra of PMV‐LA‐Na and PMV‐Na exhibited two major emission regions ranging from 450–650 nm (Figure 2F–I), with stronger emission in the long wavelength region as more LA‐NH_2_ was grafted. This was consistent with the wider absorbance in long wavelength in UV–vis diffuse reflection spectra (Figure 2D), with a reduction in average fluorescence lifetimes at 550 nm (Figure 2E and Table S1, Supporting Information).

The chemical structure of PMV‐LA‐Na was characterized by FTIR and ^1^H NMR. As seen in Figure S10, Supporting Information, the absorbance peak of C═O in the anhydride group disappeared, and a new peak emerged at 1587 cm^−1^, indicating the stretching vibration band of the COO^−^ ions in PMV‐LA‐Na films. Likewise, the absorbance of C═O in amide became more obvious at 1651 cm^−1^ with the addition of LA‐NH_2_. According to the previous report, the vinyl acetate groups would be progressively hydrolyzed, which was consistent with the weakened peak of C═O in vinyl acetate groups at 1724 cm^−1^.^[^ ^8c^ ^]^ In Figure S11, Supporting Information, the areas at *δ* = 5.0–5.2, 2.6–2.9, and 1.8–2.2 ppm belong to H (4), H (1, 2), and H (3, 5) in PMV‐Na. The hydrolyzation of vinyl acetate groups induced the moving of H (4) to H(a) with a lower frequency and the emergence of H(b) at *δ* = 1.9 ppm in the precursor solution of PMV‐LA‐Na, which implies the generation of sodium acetate after hydrolyzation. The ratio of H (4) and H (b) could be utilized to estimate the hydrolyzation ratio of vinyl acetate.

The chromophore of PMV‐LA‐Na films was investigated, and it was speculated that there are two major chromophores based on the 3D emission spectra of PMV‐LA‐Na and PMV‐Na films (Figure 2F–I). The second chromophore with a longer excitation wavelength became more apparent after the introduction of more LA‐NH_2_ (Figure S12 and Table S1, Supporting Information), during which less NaOH was utilized to neutralize the anhydride groups rather than hydrolyze the vinyl acetate groups. Referring to the previous report, without the addition of LA‐NH_2_, the second chromophores likewise became more apparent as the amount of NaOH was increased, demonstrating the critical function of ─OH in the second chromophores.^[^ ^8c^ ^]^ To control the variation, PMV‐0.1LA‐Na with different NaOH amounts were also prepared, named PMV‐0.1LA‐0.5Na and PMV‐0.1LA‐2Na, respectively (the number before Na represents the ratio to the amount of NaOH added). Surprisingly, the 3D emission spectra of PMV‐0.1LA‐0.5Na were identical to PMV‐0.1LA, suggesting the decisive roles of sufficient carboxylate group in both chromophores (Figure S13A, Supporting Information and Figure 1G). As for PMV‐0.1LA‐2Na, the second chromophores became dominant (Figure S13B, Supporting Information) due to the excessive addition of NaOH, which promotes the formation of the second chromophores through the hydrolysis of vinyl acetate groups. Moreover, the maximum emission wavelength of PMV‐0.5LA‐Na was even longer than PMV‐0.1LA‐2Na, indicating the effect of electron‐rich atoms in LA‐NH_2_ on CL (Figures S12D and S14, Supporting Information). In summary, the first chromophores consist of the aggregation of the carboxylate group, and the second chromophores are made up of the aggregation of carboxylate and hydroxyl groups. The abnormal emission region of PMV‐0.5LA‐Na is ascribed to the aggregation of electron‐rich atoms in LA‐NH_2_, as well as the carboxylate and hydroxyl group.

**Figure 3:**
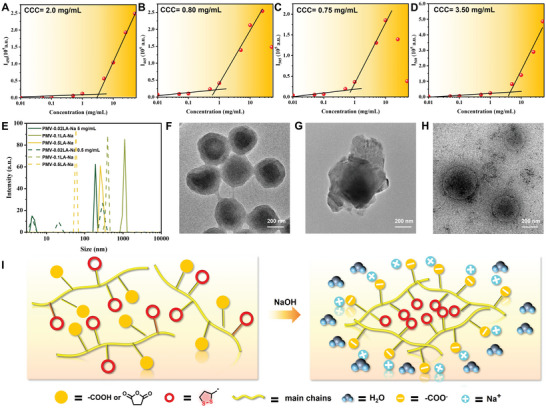
Plots of intensity versus concentration for A) PMV‐Na, B) PMV‐0.02LA‐Na, C) PMV‐0.1LA‐Na, and D) PMV‐0.5LA‐Na precursor solutions. E) Size distribution determined by DLS for PMV‐Na, PMV‐0.02LA‐Na, PMV‐0.1LA‐Na, and PMV‐0.5LA‐Na precursor solutions (concentration: 0.5 mg mL^−1^ for dotted line and the 5 mg mL^−1^ for solid line). TEM images of F) PMV‐0.02LA‐Na, G) PMV‐0.1LA‐Na, and H) PMV‐0.5LA‐Na. I) Conceptual illustration of self‐assembly mechanism.

Then, a more thorough investigation of the mechanism of ROP of a side‐chain disulfide‐containing five‐membered ring was conducted. As reported previously, the hydrophilic carboxylate groups and hydrophobic disulfide‐containing five‐membered rings would drive the self‐assembly of the amphiphilic PMV‐LA‐Na.^[^ ^10^, ^15^ ^]^ Fortunately, the dependence of CL toward the aggregation of carboxylate allowed the study of the self‐assembly of supramolecule via fluorescence spectra. The critical cluster concentration (CCC), which mirrors the critical changing of aggregation of subgroups in solution by the mutation of CL intensity, could be utilized to study the self‐assembly of supramolecule.^[^ ^16^ ^]^ Compared to PMV‐Na (2 mg mL^−1^), the CCC value of PMV‐0.02LA‐Na (0.8 mg mL^−1^) and PMV‐0.1LA‐Na (0.75 mg mL^−1^) decreased while PMV‐0.5LA‐Na (3.5 mg mL^−1^) increased (**Figure** **3**A–D). This indicates that the introduction of hydrophobic groups in PMV‐0.02LA‐Na and PMV‐0.1LA‐Na is beneficial to the aggregation of hydrophilic carboxylate and decrease the value of CCC, while excessive grafting would reduce the density of hydrophilic subgroups, leading to the increase of CCC. Besides, the variation of CCC also implies self‐assembly in the system. Then, the self‐assembly was also characterized by dynamic light scattering (DLS) measurements and transmission electron microscope (TEM). Compared to the concentration slightly below the CCC, one dominant peak with enhanced intensity could be observed in PMV‐LA‐Na with a concentration slightly above CCC (Figure 3E), which belongs to the self‐assembled microcells. And the diameters of self‐assembled microcells determined by DLS were similar to what we observed in TEM (Figure 3F–H) while the impurity of PMV‐0.5LA‐Na may be attributed to sodium acetate. The self‐assembly of PMV‐LA‐Na was also confirmed by the abnormal microstructure in SEM images (Figures S15 and S16, Supporting Information). The self‐assembly of the amphiphilic PMV‐LA‐Na with hydrophilic carboxylate groups and hydrophobic disulfide‐containing five‐membered rings could be explained by Figure 3I.

The hydrophobic five‐membered rings tend to aggregate to decrease the interaction with the surrounding high‐energy water molecules. Then, the increased intermolecular proximity would trigger the intermolecular dynamic covalent exchange of disulfide bonds, leading to the evaporation‐induced cross‐linking (EICL), which has been demonstrated by the dynamic covalent ROP of sodium thioctate after the evaporation of the solvent.^[^ ^10a^ ^]^ To further confirm this mechanism in this work, pLA‐NH_2_ was also prepared by simply evaporating the solvents of LA‐NH_2_, which could not redissolve in the original solvent but in DMSO, indicating the polymerization of dynamic covalent disulfide bonds. pLA‐NH_2_ also showed decreased absorbance at 330 nm (Figure S17A, Supporting Information), reflecting the ring opening of the disulfide‐containing five‐membered ring. Besides, considering the inhabitation of non‐radiative transition and the aggregation of subgroups after the polymerization, pLA‐NH_2_ also showed higher CL intensity after the polymerization (Figure S17B, Supporting Information).^[^ ^9a^ ^]^ The 3D emission spectra and photoluminescence spectra of pLA‐NH_2_ film (Figure S18, Supporting Information) showed similar maximum emission wavelength to PMV‐0.5LA‐Na (Table S1, Supporting Information), indicating that the electron‐rich atoms of LA‐NH_2_ also participate in the TSI of subgroups in PMV‐LA‐Na. The polymerization of LA‐NH_2_ greatly confirms that the increased intermolecular proximity during the evaporation of solvents would induce the intermolecular dynamic covalent exchange of disulfide bonds.

**Figure 4:**
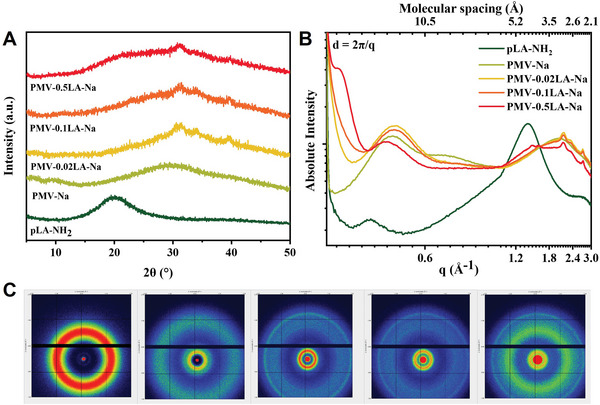
A) XRD patterns of pLA‐NH_2_, PMV‐Na, PMV‐0.02LA‐Na, PMV‐0.1LA‐Na, and PMV‐0.5LA‐Na films. B) 1D and C) 2D SAXS patterns of pLA‐NH_2_, PMV‐Na, PMV‐0.02LA‐Na, PMV‐0.1LA‐Na, and PMV‐0.5LA‐Na films.

Small angle X‐ray scattering (SAXS) and X‐ray diffractometer (XRD) were used to further characterize the chromophores and structure. As shown in **Figure** **4**A, the lack of significant diffraction peaks implied the amorphous forms of all polymers. However, with the increased addition of LA‐NH_2_, the wide diffraction peaks of pLA‐NH_2_ at 20° also appeared in PMV‐0.5LA‐Na. Further, considering the high performance of SAXS for determining the micro‐structures of polymeric materials, the SAXS was utilized to characterize the stacking of subgroups (Figure 4B,C). The sharp scattering peak at 2.03 Å^−1^ of PMV‐Na was related to the packing distance of 3.09 Å between the carboxylate group calculated by the formula in Figure 4B.^[^ ^6^, ^8^ ^]^ Likewise, the peak at 1.40 Å ^−1^ and 2.67 Å ^−1^ could also be attributed to the tight packing of the side‐chain electron‐rich atoms (4.48 Å and 2.35 Å) in pLA‐NH_2_ after the polymerization, while the peak at 20.39 Å belongs to folded conformation.^[^ ^9a^ ^]^ Although PMV‐LA‐Na films have similar diffraction peaks with PMV‐Na, with the increased addition of LA‐NH_2_, the diffraction peaks of pLA‐NH_2_ also appeared in PMV‐0.5LA‐Na. These data further verify the similar chromophores of PMV‐Na and PMV‐LA‐Na with similar packing distances of subgroups, and the variation of packing distance accounts for the abnormal CL of PMV‐0.5LA‐Na.

Furthermore, the PMV‐LA‐Na film integrates dynamic covalent disulfide bonds, ionic bonds, and hydrogen bonds of bound water making it a potential dynamic material. The self‐healing ability of PMV‐LA‐Na film was confirmed by the scratching experiments. Typically, the scratch on the surface of the film could be self‐healed at 80 °C and 70% RH after 12 h (Figure S19, Supporting Information), which is realized by the dynamic ionic bond and disulfide bond. Besides, the reversible water adsorption/release of the carboxylate group also endows the dynamic mechanical properties of PMV‐LA‐Na films.^[^ ^10a^ ^]^ In contrast, PMV‐Na without dynamic covalent disulfide bonds cross‐linking structure could easily lose its shape exposed to a 50% RH environment (Figure S20, Supporting Information). The absorption of water was also confirmed by the wide and enhanced absorbance of PMV‐0.1LA‐Na between 3000 and 3500 cm^−1^ in FTIR spectra under 90% RH (Figure S21, Supporting Information). The humidity‐induced layer expansion can be confirmed by XRD patterns with decreased diffraction angle (Figure 4A and Figure S22, Supporting Information). Therefore, the dry film of PMV‐LA‐Na exhibited robust mechanical properties with high tensile strength and Young's tensile modulus. However, the absorbance of water under high RH (70%) would destroy the ionic bond and weaken the interaction of polymer chains, resulting in lower tensile strength and Young's tensile modulus (Figure S23, Supporting Information).

**Figure 5:**
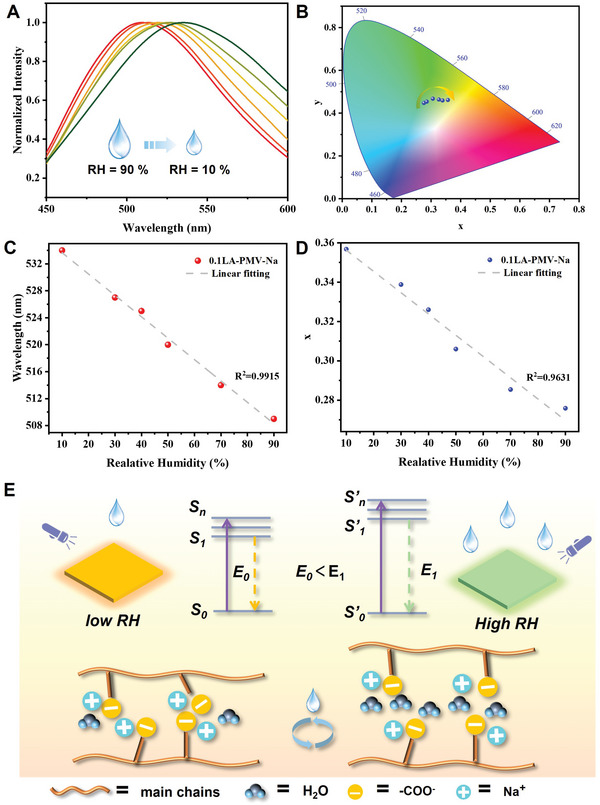
A) Normalized emission spectra of PMV‐0.1LA‐Na under varied RH excited at 370 nm. B) The CIE coordinate diagram of PMV‐0.1LA‐Na under varied RH. C) Plots and linear fit for emission wavelength and RH. D) Plots and linear fit for CIE x and RH. E) Conceptual illustration of mechanism.

Generally, CL depends greatly on the aggregation and the TSI of subgroups. Since the CL of PMV‐LA‐Na originates from the aggregation of the carboxylate group, we first measured the photoluminescence spectra of PMV‐Na solids and their aqueous solutions with large differences in aggregation distance. Compared to the aqueous solutions of PMV‐Na, the closer aggregation of the carboxylate group in PMV‐Na solids induced the red‐shift of its maximum emission wavelength from 500 to 540 nm excited at 370 nm (Figure S24, Supporting Information). Subsequently, a drop of water was added to the solid sample to increase the aggregation distance, and a significant blue shift from 540 to 505 nm was also observed. The above results indicate the possibility of achieving responsive fluorescence by changing the aggregation distance of the carboxylate group. Then, considering the reversible water adsorption/release property of the carboxylate group in cross‐linking structure, the CL of PMV‐0.1LA‐Na film under different RH was recorded. The maximum emission wavelength of PMV‐0.1LA‐Na blue‐shifted from 534 to 508 nm along with the changes in CIE 1931 coordinates (**Figure** **5**A,B). The variation of emission wavelength and x value of CIE 1931 coordinates showed a good relationship with RH (Figure 5C,D), which was also verified by what we observed under UV light (Figure S25, Supporting Information). The emission wavelength of PMV‐0.1LA‐Na was stable after several times recycling (Figure S26, Supporting Information). And the mechanism of dynamic CL can be explained in Figure 5E. The absorbance of water would destroy the ionic bond and increase the distance between the carboxylate group, which is similar to the increased distance in PMV‐Na solutions. Since the TSI needs the aggregation of subgroups, the larger distance of the carboxylate group could weaken the interactions between subgroups and broaden the energy gap between HOMO and LOMO, resulting in a blue shift of emission wavelength. Conversely, the release of water under a low RH environment would enhance the ionic bond and narrow the energy gap, resulting in a red shift of emission wavelength. In conclusion, making use of the intrinsic properties of CL could provide a new strategy for constructing stimuli‐responsive material.

**Figure 6:**
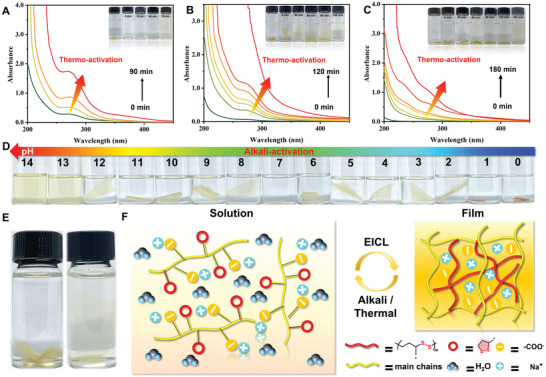
UV–vis absorption spectra of A) PMV‐0.02LA‐Na, B) PMV‐0.1LA‐Na, and C) PMV‐0.5LA‐Na films in water with different thermal‐activation times. (Insets: Photographs of PMV‐LA‐Na films with different thermal‐activation times) D) Photographs of PMV‐0.1LA‐Na under varied pH. E) Photographs of the swelling rPMV‐0.1LA‐Na in deionized water. F) Conceptual illustration of reprocessing mechanism.

The dynamic covalent disulfide bonds and ionic bonds could also enable the recycling of the film, overcoming the shortcomings of traditional cross‐linking structures.^[^ ^17^ ^]^ Thermo‐ and alkali‐activation depolymerization of dynamic covalent disulfide bonds was achieved through heating at 80 °C and immersion in NaOH aqueous. Real‐time UV–vis absorption spectroscopy was performed to detect the thermo‐activation depolymerization. As shown in **Figure** **6**A–C, the enhanced absorbance of PMV‐LA‐Na was ascribed to the depolymerization of dynamic covalent disulfide bonds, and with the increased addition of LA‐NH_2_, more times was required to completely dissolve the cross‐linking film. Meanwhile, only high pH (greater than 13) could activate the dynamic covalent disulfide bonds, showing the balance of reprocessability and stability during application (Figure 6D). Considering that the heating process was relatively simple and convenient, the reprocessed film of PMV‐0.1LA‐Na was prepared and the ^1^H NMR spectra were utilized to characterize the changing of chemical structure. The reprocessed PMV‐0.1LA‐Na had similar ^1^H NMR spectra to the original sample, with more hydrolyzation of vinyl acetate groups, which was inevitable during the heating process (Figure S27, Supporting Information). After the evaporation of water, the obtained rPMV‐0.1LA‐Na film could also resist the water with slightly decreased mechanical properties, implying the recross‐linking of dynamic covalent disulfide bonds (Figure 6E and Figure S23C, Supporting Information). The dynamic covalent disulfide bonds could be activated by alkali or thermal and became recross‐linking after the EICL (Figure 6F). As for its CL, the second chromophores became dominant due to the hydrolyzation of vinyl acetate (Figure S28, Supporting Information). In a sense, apart from the grafting ratio of LA‐NH_2_, the reprocessing methods could also affect or adjust the CL of PMV‐LA‐Na.

## 3 Conclusion

In this work, dynamic supramolecular films were designed and synthesized by the self‐assembly of hydrophilic carboxylate chromophores and hydrophobic disulfide‐containing five‐membered rings. The self‐assembly was verified by the changing of CCC in CL, which is relevant to the aggregation state of carboxylate. Further characterizations reveal the intermolecular dynamic covalent exchange of gathering disulfide bonds even in amphiphilic supramolecules. Then, the water‐sensitive carboxylate chromophores could easily change the aggregation distance upon the stimuli of surrounding water vapor and vary the TSI and CL with different emission wavelengths. The ROP of dynamic covalent allows the supramolecules to maintain stability in the response process and endow it with self‐healing and reprocessing properties. This work not only achieves the monitoring of self‐assembly and transforms the water vapor stimuli to visual CL, but provides a new strategy to utilize the intrinsic properties of CL and widens the applications of CL.

## 4 Experimental Section

### Materials

Maleic anhydride (MAh, 99% purity), 2,2‐azobisisobutyronitrile (AIBN, 99%), vinyl acetate (VAc 99%), *n*‐butyl acetate, *α*‐lipoic acid (LA, 99% purity), 1,1′‐carbonyldiimidazole (CDI, 99% purity), ethylenediamine (99% purity) and other solvents were all purchased from Aladdin‐reagent Inc.

### Self‐Stabilized Precipitation Polymerization

PMV was synthesized by self‐stabilized precipitation polymerization reported previously.^[^ ^8b^ ^]^ (*M*_n_ = 44 004, *M*_w_ = 110 107, *M*_w_/*M*_n_ = 2.502)

### Synthesis of LA‐NH_2_

LA‐NH_2_ was synthesized by a similar method reported previously.^[^ ^11a^ ^]^ First, 2.00 g LA and 1.88 g CDI were added to anhydrous chloroform (10 mL) and stirred for 30 min under N_2_. The solution was added dropwise into ethylenediamine (3.34 mL) in anhydrous chloroform (20 mL) at 4 °C under N_2_ and kept for 12 h at room temperature. The product was washed with brine and NaOH aqueous solution (10 mm) three times. And the organic phase was dried by Na_2_SO_4_ before the removal of solvents. The final product was yellow oil (2.23 g, 83.6%).

### Synthesis of pLA‐NH_2_

The obtained LA‐NH_2_ was concentrated to ≈15 mL in CHCl_3_. Then, the solution was left in the air to allow slow evaporation of the solvents.

Synthesis of PMV‐LA: 0.5 PMV was dissolved in 10 mL acetone. And LA‐NH_2_ dissolved in acetone was added to the PMV solution. The solution was purged with N_2_ and heated at 50 °C for 12 h. Then, the mixture was poured into petroleum ether to precipitate PMV‐LA. The polymer was redissolved in acetone and precipitated in petroleum ether three times to remove the impurity. Last, the products were vacuum dried at 50 °C to constant weights.

### Preparation of the PMV‐LA‐Na Film

0.43 g NaOH was dissolved in 20 mL deionized water, and 1.0 g PMV‐LA was added. The mixture was stirred overnight until PMV‐LA was completely dissolved. The water was then slowly evaporated by leaving the solution in the air. After 2–3 days, PMV‐0.02LA‐Na, PMV‐0.1LA‐Na, and PMV‐0.5LA‐Na films with thicknesses of 0.298, 0.270, and 0.285 cm were obtained. PMV‐Na film with thicknesses of 0.267 cm was prepared by a similar method.

### Characterization

^1^H NMR spectra were recorded on a Bruker Avance spectrometer with acetone‐d6, DMSO‐d6, and D_2_O. FTIR spectra were obtained on an FTIR spectrometer (Nicolet 6700, USA). The molecular weights (*M*_w_) and polydispersity (*M*_w_/*M*_n_) of polymers were recorded on a Waters gel permeation chromatography (HLC‐8320GPC, Japan) using H_2_O/0.1 m NaNO_3_ as eluent. UV absorption spectra were taken on a UV–vis spectrophotometer (TU‐1950, China). The UV–vis diffuse reflection spectra were performed on a UV/VIS/NIR spectrophotometer (UV‐3600 plus, Japan). Photoluminescence spectra were measured on a fluorescence spectrometer (FS5, UK). Decay times were conducted on Lifespec ll (Edinburgh, UK). The absolute quantum yields were conducted on FLS‐1000 (Edinburgh, UK). XRD patterns were recorded using an XRD analyzer (Bruker‐D8, Germany). SAXS was obtained on a 2D multifunctional small Angle X‐ray scatterer (Xeuss 3.0HR, France). The morphology of the polymer was observed by a scanning electron microscope (Hitachi S‐4800). The TEM images were obtained on JEM‐2100plus. Thermogravimetric analysis was carried out on PerkinElmer with a heating rate of 10 °C min^−1^. The stress–strain curves were recorded with a tensile tester (Instron 5967, United States). The film was shaped as a rectangle sample (20 × 10 × 0.5 mm). The tested polymer films were preplaced for at least 2 h under the given RH to reach the adsorption/desorption equilibrium with water. DLS was performed on a Malvern Nano‐ZS. Dynamic mechanical analysis (DMA) measurements were performed on a DMA Q800 with a temperature range from 0 to 100 °C at a rate of 3 °C min^−1^ in tension mode with a frequency of 1 Hz. The self‐healing was recorded by using a VHX‐1000C.

### Statistical Analysis

The quantitative data were presented as mean ± standard deviation (s. d.) from at least three measurements. Normalization treatment was performed on the 3D emission spectra of solids.

## Conflict of Interest

The authors declare no conflict of interest.

## Supporting information

Supporting Information

## Data Availability

Research data are not shared.
