# Supplementary material for: A Self‐Assemble Supramolecular Film with Humidity Visualization Enabled by Clusteroluminescence
Source: Adv Sci (Weinh). 2023 Nov 9;11(1):2304946. doi: 10.1002/advs.202304946 (PMC10767432; doi:10.1002/advs.202304946)
Supplement: Supplementary file 1 — Supporting Information [file ADVS-11-2304946-s001.pdf]

ADVANCED SCIENCE
Open Access

Supporting Information

for *Adv. Sci.*, DOI 10.1002/advs.202304946

A Self-Assemble Supramolecular Film with Humidity Visualization Enabled by Clusteroluminescence

Xiang Chen, Chenxi Hu, Yang Wang, Ting Li, Jie Jiang, Jing Huang, Shibo Wang, Weifu Dong* and Jinliang Qiao**

Supporting Information

Dynamic self-assemble supramolecular films with Humidity Visualization Enabled by Clusteroluminescence

Xiang Chen,[1] Chenxi Hu,[2] Yang Wang,[1] Ting Li,[1] Jie Jiang,[1] Jing Huang,[1] Shibo Wang,[1] Weifu Dong*,[1] Jinliang Qiao*[2]*

Corresponding Authors:

Yang Wang: ywang@jiangnan.edu.cn

Weifu Dong: wfdong@jiangnan.edu.cn

Jinliang Qiao: qiaojl.bjhy@sinopec.com

Tel.: +86-510-8532-6290.

[1]The Key Laboratory of Synthetic and Biological Colloids, Ministry of Education, School of Chemical and Material Engineering, Jiangnan University, 1800 Lihu Road, Wuxi 214122, China;

[2]SINOPEC, Beijing Research Institute of Chemical Industry, Beijing, 100013, China.

Table of Contents

Figure S1. FTIR spectra of LA and LA-NH_2. 2
Figure S2. ^{1}H NMR spectra of LA-NH_2.......... 3
Figure S3. FTIR spectra of PMV and PMV-LA solids 3
Figure S4. ^{1}H NMR spectra of PMV and PMV-LA solids 3
Figure S5. Photographs of PMV and PMV-LA solids under natural and UV light. 4
Figure S6. Photoluminescent spectra of (A) PMV, (B) PMV-0.02LA, (C) PMV-0.1LA and (D) PMV-0.5LA solids 4
Figure S7. Decay times spectra of PMV, PMV-0.02LA, PMV-0.1LA and PMV-0.5LA solids excited at 485 nm. 5
Figure S8. Photographs of (A) PMV-0.02LA-Na, (B) PMV-0.1LA-Na and (C) PMV-0.5LA-Na film in water. 5
Figure S9. Temperature dependence of (A) tan δ and (B) E′ for pLA-NH2, PMV-Na, PMV-0.02LA-Na, PMV-0.1LA-Na and PMV-0.5LA-Na at 1 Hz. (C) TGA and (D) DTG curves of PMV-Na, PMV-0.02LA-Na, PMV-0.1LA-Na and PMV-0.5LA-Na 6
Figure S10. FTIR spectra of PMV-Na, PMV-0.02LA-Na, PMV-0.1LA-Na and PMV-0.5LA-Na films. 6
Figure S11. ^{1}H NMR spectra of PMV-Na, PMV-0.02LA-Na, PMV-0.1LA-Na and PMV-0.5LA-Na precursor solution 7

Figure S12. Photoluminescent spectra of (A) PMV-Na, (B)PMV-0.02LA-Na, (C)PMV-0.1LA-Na and (D)PMV-0.5LA-Na films 7
Figure S13. Normalized 3D photoluminescence spectra of (A) PMV-0.1LA-0.5Na and (B) PMV-0.1LA-2Na 8
Figure S14. Photoluminescence spectra of PMV-0.1LA-2Na 8
Figure S15. SEM images of PMV-Na. 8
Figure S16. SEM images of (A) PMV-0.02LA-Na, (B) PMV-0.1LA-Na and (C) PMV-0.5LA-Na (insets: SEM images with different magnifications). 8
Figure S17. (A) UV-Vis absorption spectra and (B) photoluminescent spectra of LA-NH_2 and pLA-NH_2 solutions (5 mg/mL in DMSO). 9
Figure S18. Normalized 3D photoluminescence spectra and photoluminescent spectra of pLA-NH_2. 9
Figure S19. Optical microscopy images of damaged and healed films at 80 °C and 70% RH 9
Figure S20. Photographs of PMV-Na at 50% RH. 9
Figure S21. FTIR spectra of PMV-0.1LA-Na at different RH. 10
Figure S22. XRD patterns of PMV-0.1LA-Na at 90% RH. 10
Figure S23. Photographs of PMV-0.02LA-Na under UV light. (B) Strain-stress curves of pLA-NH_2, PMV-Na, PMV-0.02LA-Na, PMV-0.1LA-Na and PMV-0.5LA-Na at 30% RH. (C) Strain-stress curves of PMV-0.02LA-Na, PMV-0.1LA-Na, PMV-0.5LA-Na and rPMV-0.1LA-Na at 70% RH. 11
Figure S25. Photographs of PMV-0.1LA-Na at different RH under natural light and UV light. 11
Figure S26. Emission wavelength of PMV-0.1LA-Na under 90% and 10% RH with different cycle times. 12
Figure S27. ^{1}H NMR spectra of rPMV-0.1LA-Na. 12
Figure S28. Normalized 3D emission spectra of rPMV-0.1LA-Na. 12
Table S1. Optical properties of PMV-0.02LA, PMV-0.02LA, PMV-0.5LA, PMV-Na, PMV-0.02LA-Na, PMV-0.1LA-Na, PMV-0.5LA-Na, PMV-0.1LA-0.5Na, PMV-0.1LA-2Na, rPMV-0.1LA-Na and pLA-NH_2. 13

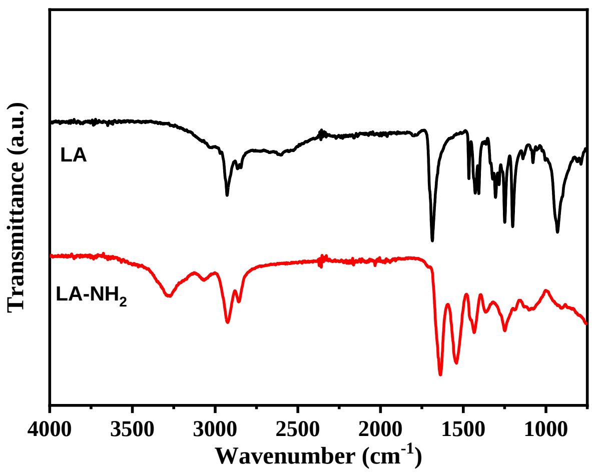

Figure S1. FTIR spectra of LA and LA-NH_2.

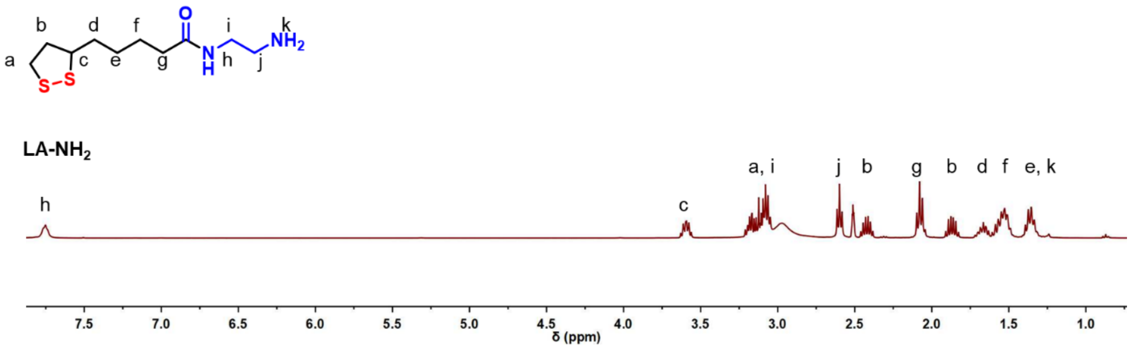

Figure S2. ^{1}H NMR spectra of LA-NH_2.

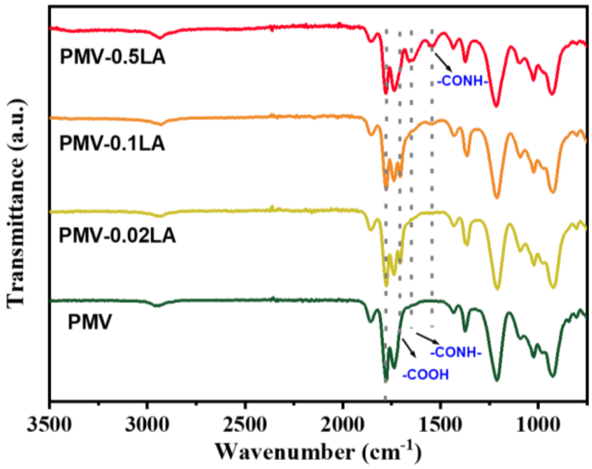

Figure S3. FTIR spectra of PMV, PMV-0.02LA, PMV-0.1LA and PMV-0.5LA solids

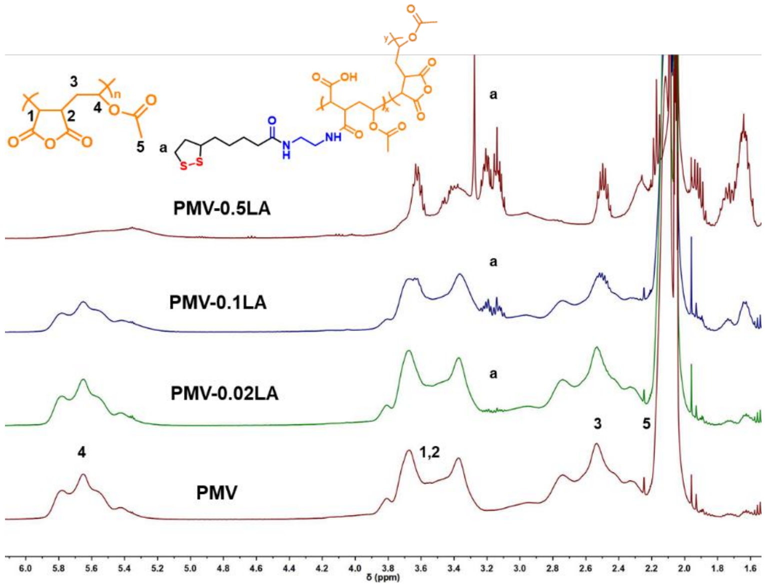

Figure S4. ^{1}H NMR spectra of PMV, PMV-0.02LA, PMV-0.1LA and PMV-0.5LA solids

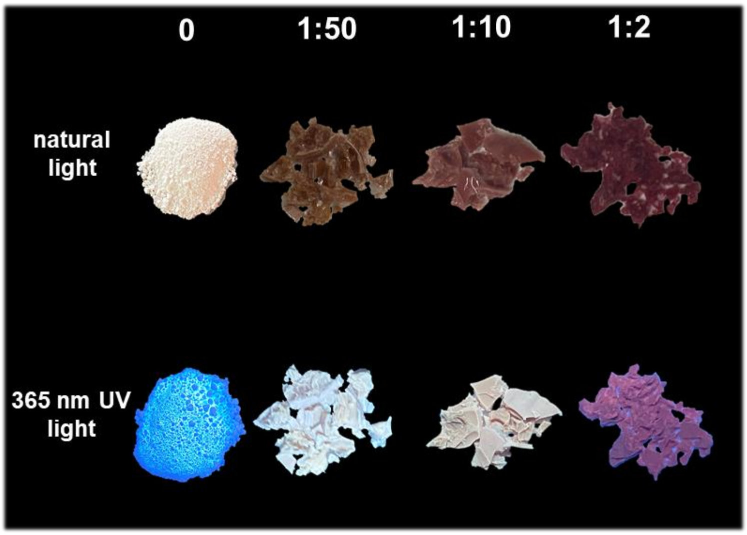

Figure S5. Photographs of PMV, PMV-0.02LA, PMV-0.1LA and PMV-0.5LA solids under natural and UV light.

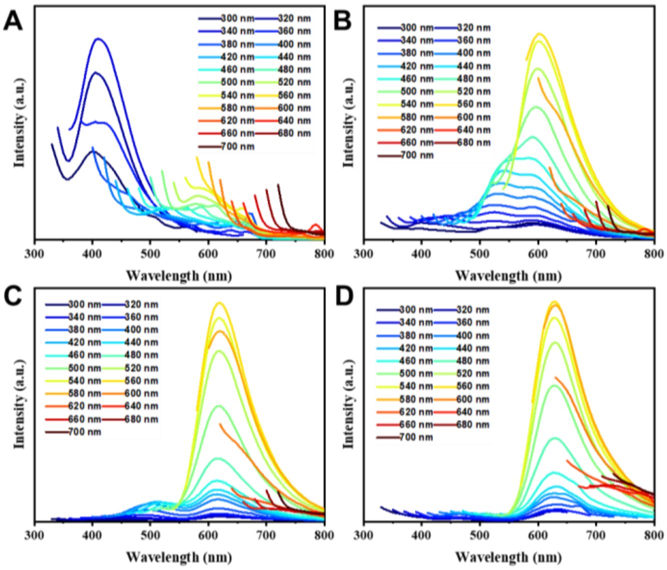

Figure S6. Photoluminescent spectra of (A) PMV, (B) PMV-0.02LA, (C) PMV-0.1LA and (D) PMV-0.5LA solids

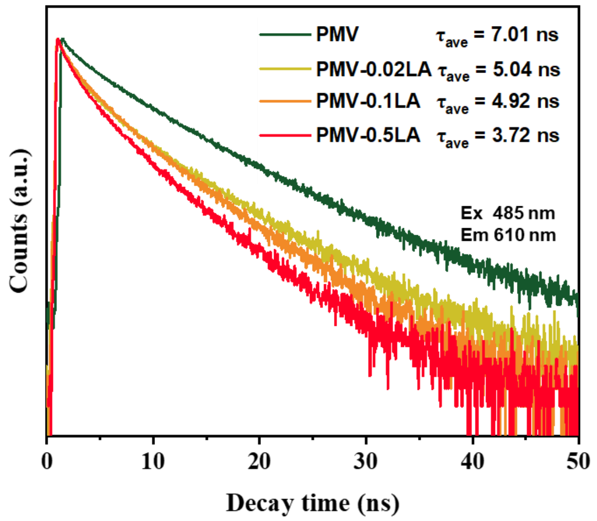

Figure S7. Decay times spectra of PMV, PMV-0.02LA, PMV-0.1LA and PMV-0.5LA solids excited at 485 nm.

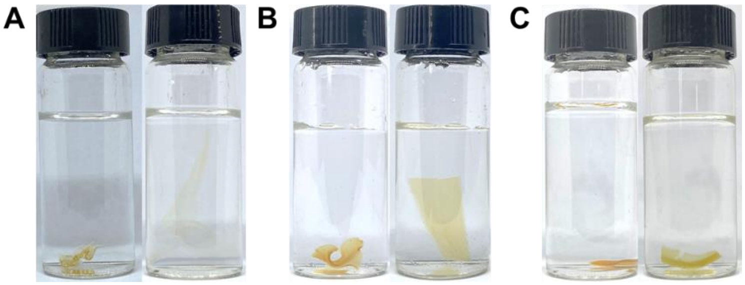

Figure S8. Photographs of (A) PMV-0.02LA-Na, (B) PMV-0.1LA-Na and (C) PMV-0.5LA-Na film in water.

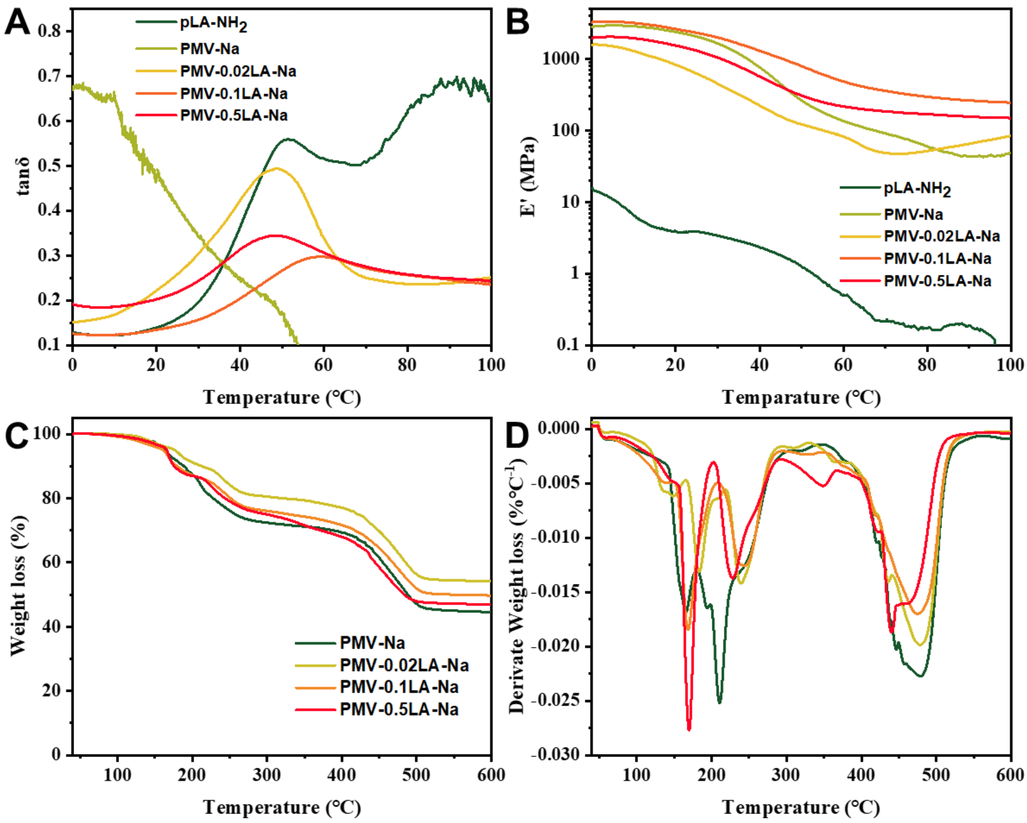

Figure S9. Temperature dependence of (A) tan δ and (B) E′ for pLA-NH_2, PMV-Na, PMV-0.02LA-Na, PMV-0.1LA-Na and PMV-0.5LA-Na at 1 Hz. (C) TGA and (D) DTG curves of PMV-Na, PMV-0.02LA-Na, PMV-0.1LA-Na and PMV-0.5LA-Na

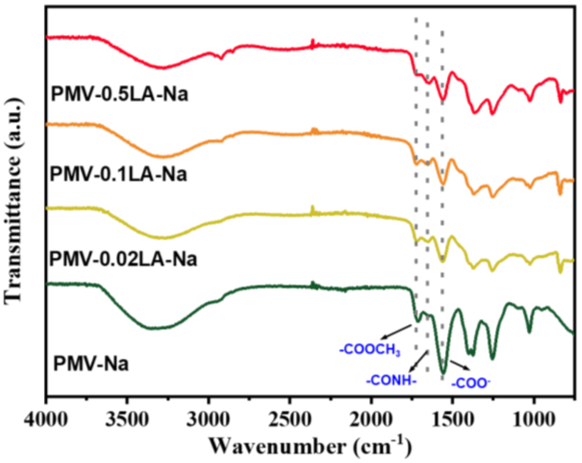

Figure S10. FTIR spectra of PMV-Na, PMV-0.02LA-Na, PMV-0.1LA-Na and PMV-0.5LA-Na films.

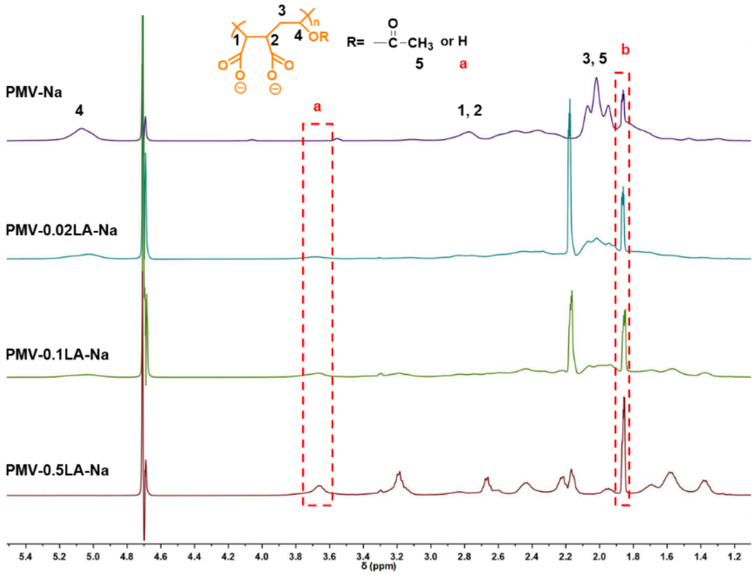

Figure S11. ^{1}H NMR spectra of PMV-Na, PMV-0.02LA-Na, PMV-0.1LA-Na and PMV-0.5LA-Na precursor solution.

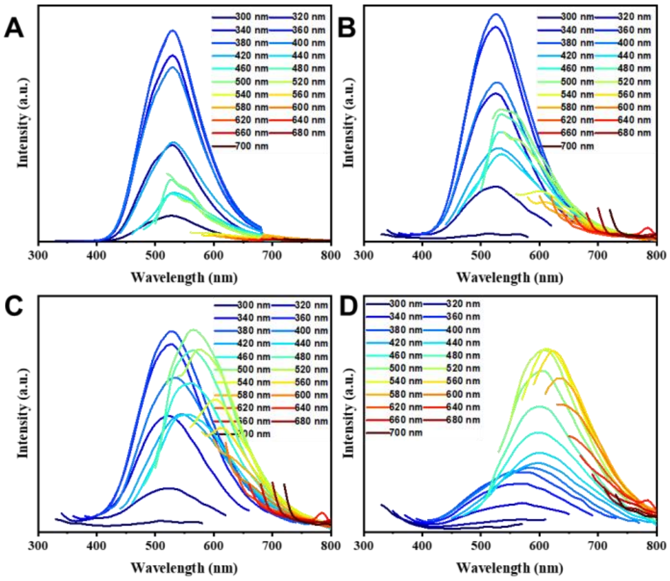

Figure S12. Photoluminescent spectra of (A) PMV-Na, (B)PMV-0.02LA-Na, (C)PMV-0.1LA-Na and (D)PMV-0.5LA-Na films

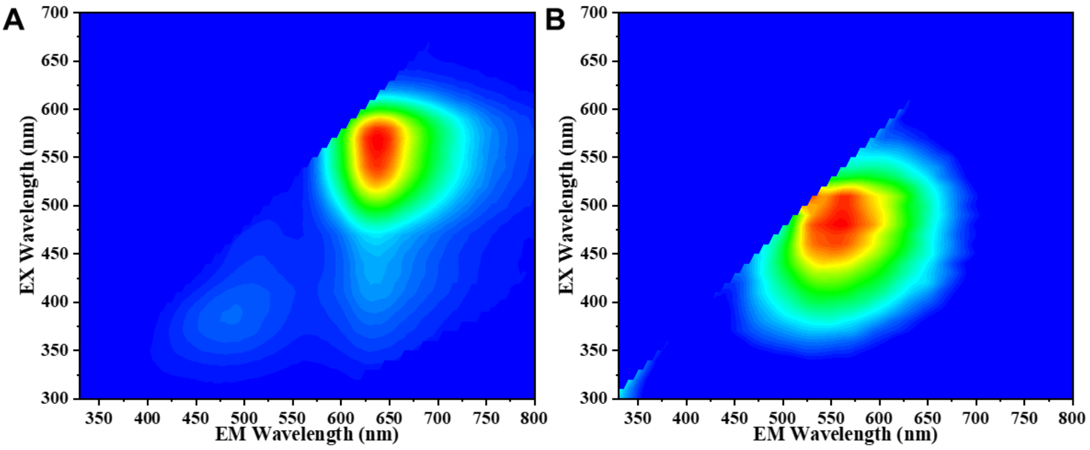

Figure S13. Normalized 3D photoluminescence spectra of (A) PMV-0.1LA-0.5Na and (B) PMV-0.1LA-2Na

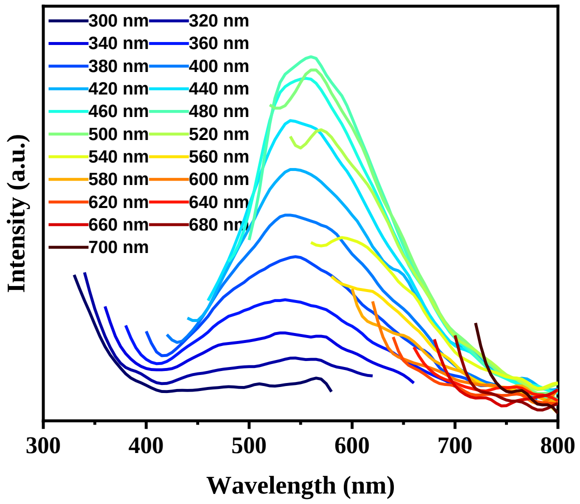

Figure S14. Photoluminescence spectra of PMV-0.1LA-2Na films.

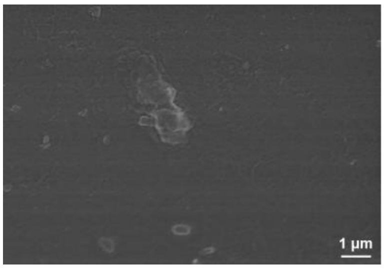

Figure S15. SEM image of PMV-Na.

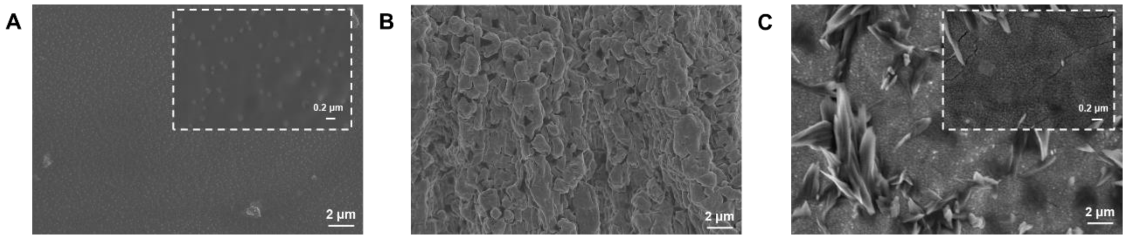

Figure S16. SEM images of (A) PMV-0.02LA-Na, (B) PMV-0.1LA-Na and (C) PMV-0.5LA-Na (insets: SEM images with different magnifications).

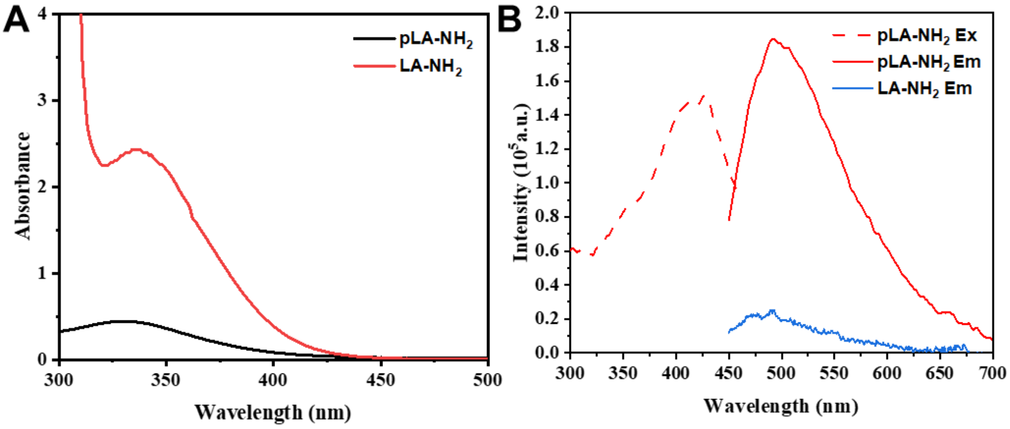

Figure S17. (A) UV-Vis absorption spectra and (B) photoluminescent spectra of LA-NH_2 and pLA-NH_2 solutions (5 mg/mL in DMSO).

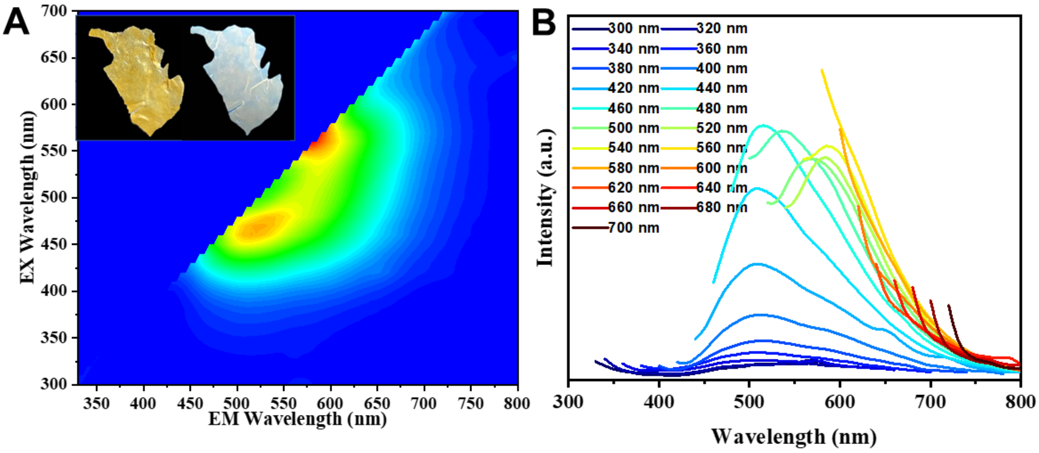

Figure S18. Normalized 3D photoluminescence spectra and photoluminescent spectra of pLA-NH_2.

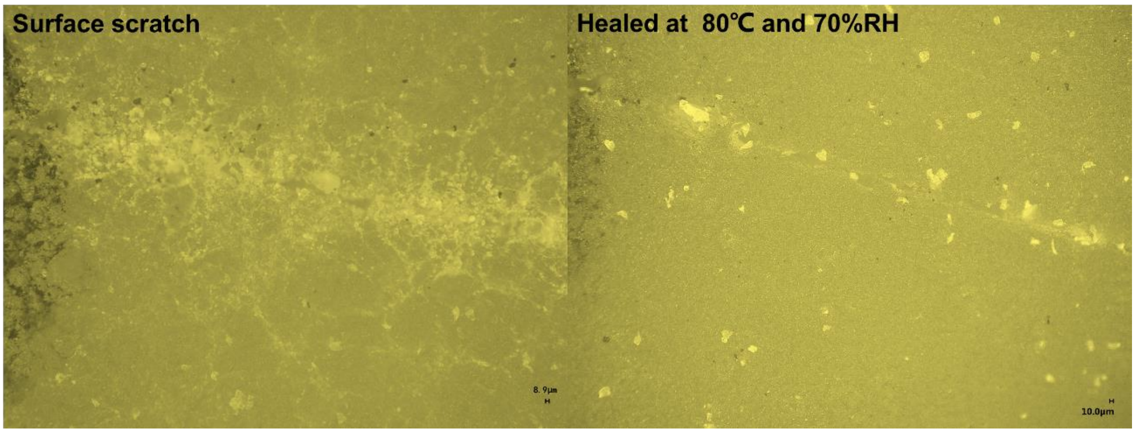

Figure S19. Optical microscopy images of damaged and healed films at 80 °C and 70% RH

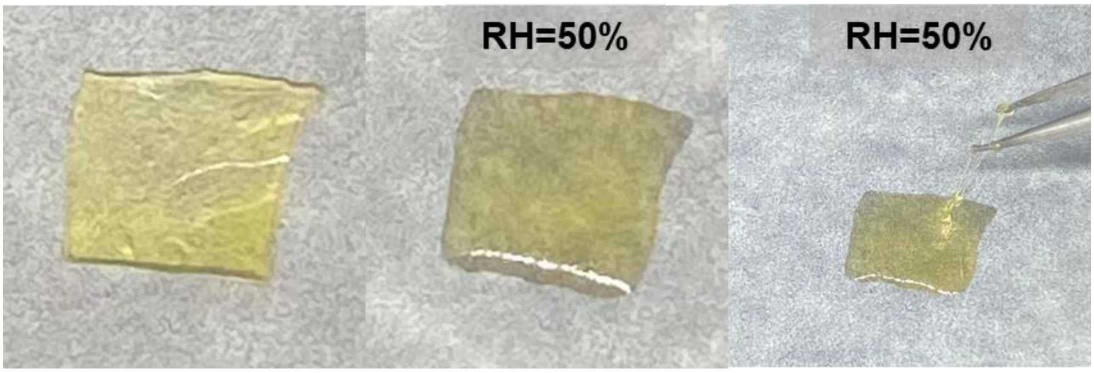

Figure S20. Photographs of PMV-Na under 50% RH.

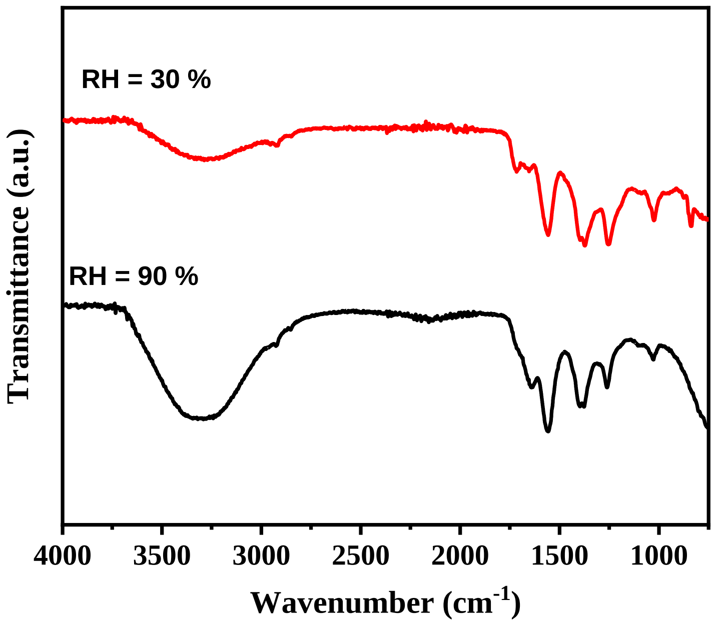

Figure S21. FTIR spectra of PMV-0.1LA-Na under different RH.

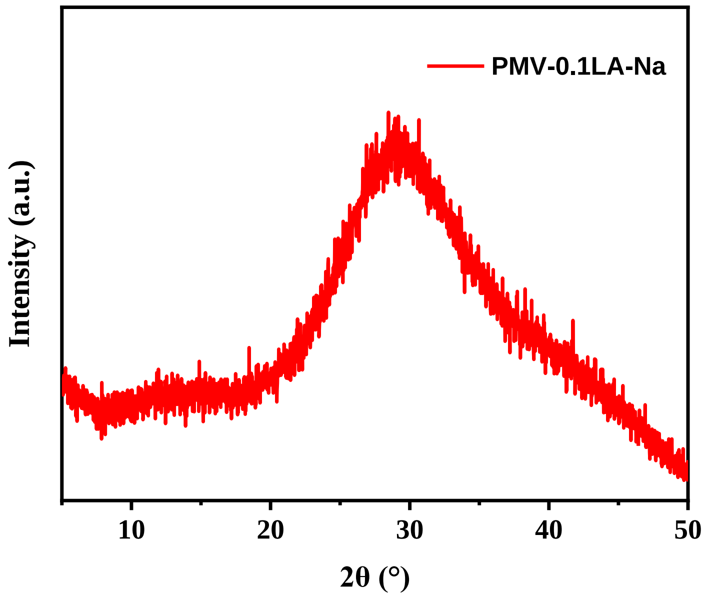

Figure S22. XRD patterns of PMV-0.1LA-Na under 90% RH.

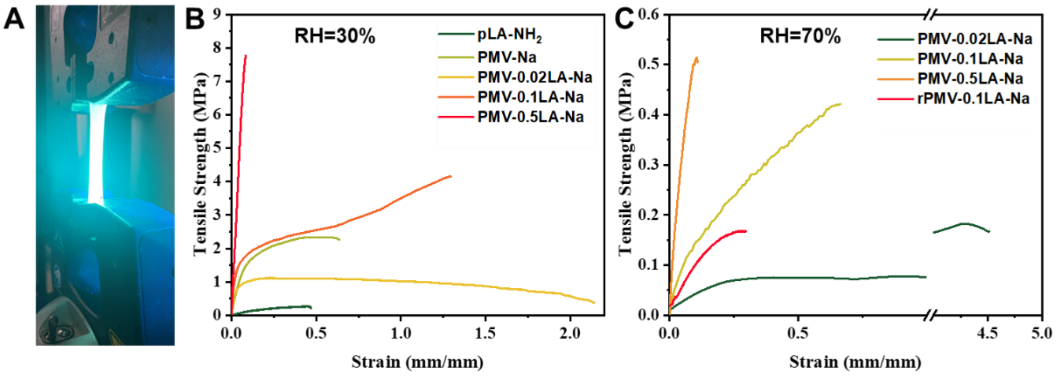

Figure S23. Photographs of PMV-0.02LA-Na under UV light. (B) Strain-stress curves of pLA-NH_2, PMV-Na, PMV-0.02LA-Na, PMV-0.1LA-Na and PMV-0.5LA-Na under 30% RH. (C) Strain-stress curves of PMV-0.02LA-Na, PMV-0.1LA-Na, PMV-0.5LA-Na and rPMV-0.1LA-Na at 70% RH.

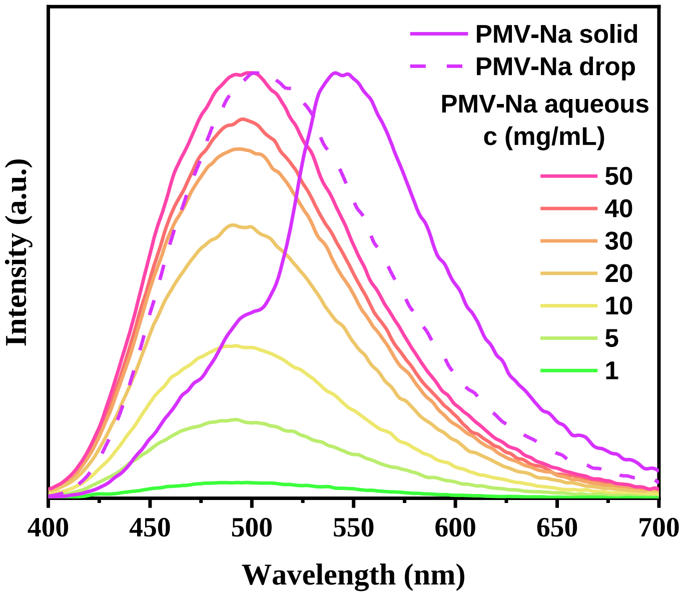

Figure S24. Photoluminescence spectra of aqueous PMV-Na, PMV-Na solids (normalized) and PMV-Na solids with a drop of water (normalized) excited at 370 nm.

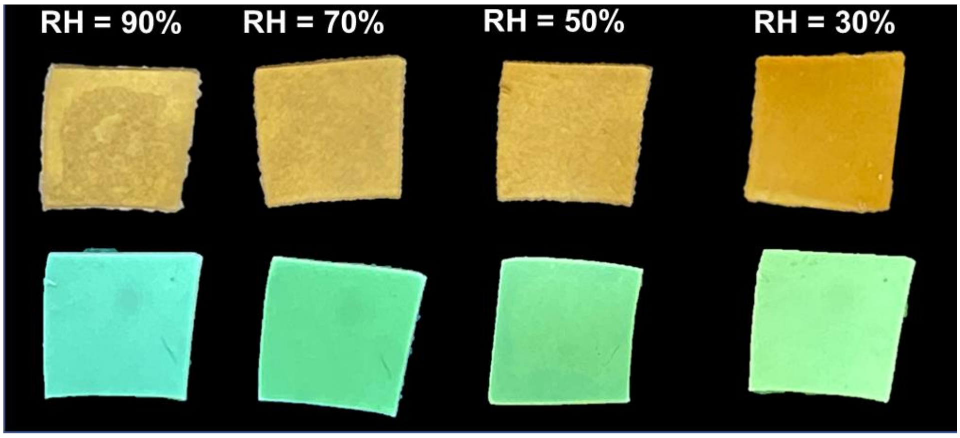

Figure S25. Photographs of PMV-0.1LA-Na at different RH under natural light and UV light.

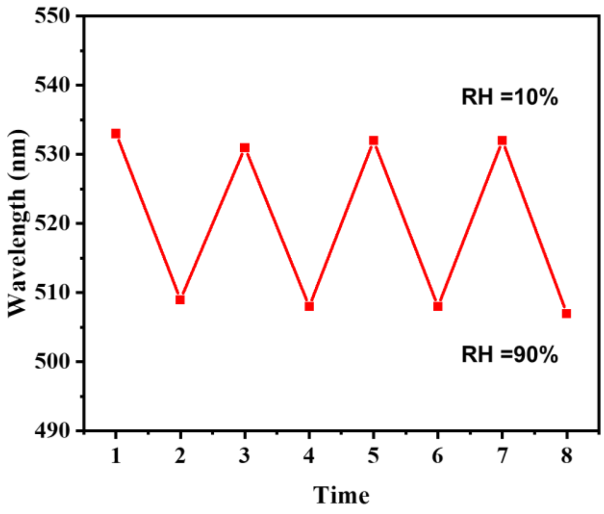

Figure S26. Emission wavelength of PMV-0.1LA-Na under 90% and 10% RH with different cycle times.

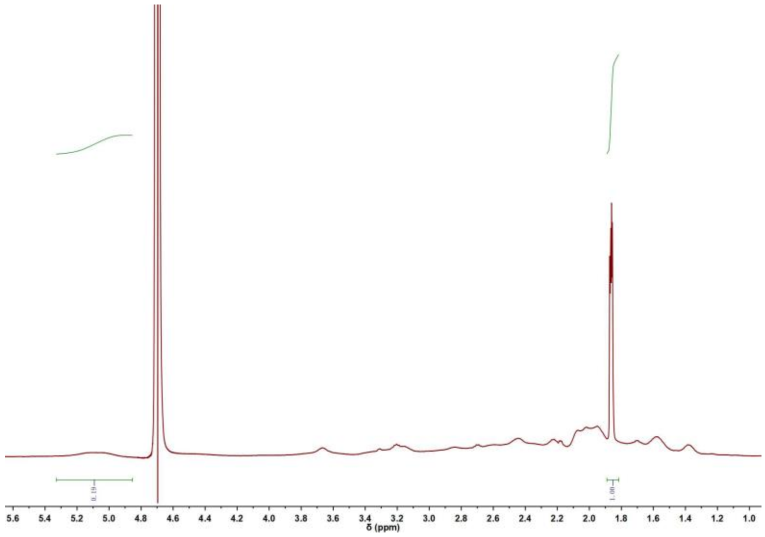

Figure S27. ^{1}H NMR spectra of rPMV-0.1LA-Na.

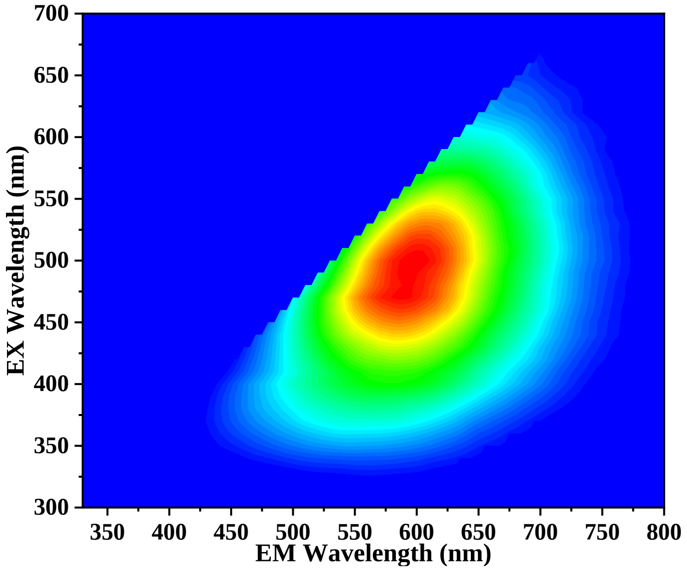

Figure S28. Normalized 3D emission spectra of rPMV-0.1LA-Na.

Table S1. Optical properties of PMV-0.02LA, PMV-0.1LA, PMV-0.5LA, PMV-Na, PMV-0.02LA-Na, PMV-0.1LA-Na, PMV-0.5LA-Na, PMV-0.1LA-0.5Na, PMV-0.1LA-2Na, rPMV-0.1LA-Na and pLA-NH_2.

Sample	λ_{ex}(nm)	λ_{em}(nm)	QY(%)	τ(ns)
PMV-0.02LA	560	610	8.59	5.04
PMV-0.1LA	560	620	8.67	4.92
PMV-0.5LA	570	630	6.02	3.72
PMV-Na	370	530	17.60	2.72
PMV-0.02LA-Na	370	530	10.40	2.48
PMV-0.1LA-Na	510	565	5.26	2.26
PMV-0.5LA-Na	550	620	5.10	2.02
PMV-0.1LA-0.5Na	570	630	7.97	3.53
PMV-0.1LA-2Na	480	560	5.40	2.03
rPMV-0.1LA-Na	500	595	5.06	2.67
pLA-NH_2	550	620	8.18	2.92
